# Statistical Speech Segmentation and Word Learning in Parallel: Scaffolding from Child-Directed Speech

**DOI:** 10.3389/fpsyg.2012.00374

**Published:** 2012-10-01

**Authors:** Daniel Yurovsky, Chen Yu, Linda B. Smith

**Affiliations:** ^1^Department of Psychology, Stanford UniversityStanford, CA, USA; ^2^Department of Psychological and Brain Sciences and Program in Cognitive Science, Indiana UniversityBloomington, IN, USA

**Keywords:** statistical learning, speech segmentation, word learning, child-directed speech, frequent frames

## Abstract

In order to acquire their native languages, children must learn richly structured systems with regularities at multiple levels. While structure at different levels could be learned serially, e.g., speech segmentation coming before word-object mapping, redundancies across levels make parallel learning more efficient. For instance, a series of syllables is likely to be a word not only because of high transitional probabilities, but also because of a consistently co-occurring object. But additional statistics require additional processing, and thus might not be useful to cognitively constrained learners. We show that the structure of child-directed speech makes simultaneous speech segmentation and word learning tractable for human learners. First, a corpus of child-directed speech was recorded from parents and children engaged in a naturalistic free-play task. Analyses revealed two consistent regularities in the sentence structure of naming events. These regularities were subsequently encoded in an artificial language to which adult participants were exposed in the context of simultaneous statistical speech segmentation and word learning. Either regularity was independently sufficient to support successful learning, but no learning occurred in the absence of both regularities. Thus, the structure of child-directed speech plays an important role in scaffolding speech segmentation and word learning in parallel.

## Introduction

Human language is richly structured, with important regularities to be learned at multiple levels (Kuhl, 2004). For instance, the human vocal apparatus can produce a staggering variety of sounds distinguishable from each other by prelinguistic infants (Eimas et al., 1971). However, only a tiny fraction of these become meaningful units – phonemes – within a particular language. Similarly, these phonemes can be strung together into an infinite number of sequences, but only a tiny fraction of these are words. Thus, infants must also solve the problem of parsing a continuous sequence of phonemes into word units. Further, some of these words refer to objects in the visual world, and so, for these segmented words, infants must solve the word-world mapping problem. In addition, speakers may refer to the same object with different words in different contexts, and different word orderings and stress patterns can radically alter an utterance’s meanings, so children must organize sounds, segments, and meanings at the levels pragmatics, syntax, and prosody as well.

An emerging theoretical consensus is that many or even all of these problems may be solved through a process of statistical learning – tracking predictive relationships between elemental units (although, cf. Marcus, 2000; Waxman and Gelman, 2009). In order to determine their native language phonemes, infants may track the distribution of tonal and formant frequencies in their input (Maye et al., 2002; Pierrehumbert, 2003). Similarly, infants may learn word boundaries by tracking sequential syllable statistics (Saffran et al., 1996), learn word-world mappings by tracking word-object occurrence statistics (Smith and Yu, 2008; Vouloumanos and Werker, 2009), and learn grammar by tracking sequential and non-adjacent dependencies between word types (Gómez and Gerken, 2000; Saffran et al., 2008). Because statistical learning at each level assumes the availability of primitives at the level below and shows how to arrive at primitives for the level above, a complete statistical account of language learning must bridge these levels. Therefore, a critical question for statistical theories of language acquisition is how learners connect these primitives.

One possibility is that the infants learn each level sequentially, proceeding from the bottom up. Learning at each level would build the units over which the next level operates, and thus higher levels would have to wait until (at least some of) the primitives at the lower levels had been acquired. This hypothesis is intuitive, and makes several predictions consistent with the extant literature. First, it predicts a developmental trajectory in statistical learning abilities: phoneme learning should come first, followed by speech segmentation, followed by word-world mapping, followed by syntax. Indeed, this is the general trend observed in infant statistical learning experiments. At 6 months, infants show sensitivity to phoneme distributions (Maye et al., 2002), at 8 months they can segment continual speech into words (Saffran et al., 1996), at 12 months they can map words onto objects using co-occurrence information (Smith and Yu, 2008), and at 18 months they can learn non-adjacent syntactic dependencies (Gómez, 2002). Second, this account predicts that infants should be able to extract regularities at one level, and use them subsequently to learn at the next higher level. This has been confirmed by recent empirical findings from Saffran and colleagues (Graf Estes et al., 2007; Hay et al., 2011) showing that statistically coherent word segments extracted from continuous speech subsequently act as superior labels in subsequent word learning. It is also supported by recent computational models showing that regularities at multiple levels can be learned serially from child-directed speech (Yu et al., 2005; Christiansen et al., 2009; Räsänen, 2011).

Alternatively, learners could acquire structure at each level in parallel. Because regularities at each level are statistically inter-related, partial acquisition of the structure at any level would reduce ambiguity at every other level (Feldman et al., 2009; Johnson et al., 2010). However, this aggregate ambiguity reduction comes at a cost: if units are uncertain at every level, demands on attention and memory are likely to skyrocket. Thus, an abundance of structure helpful for ideal learners might easily overload cognitively constrained statistical learners (Fu, 2008; Frank et al., 2010). This tradeoff is evident in recent experiments investigating simultaneous statistical speech segmentation and word learning. In these experiments, adult learners engaged in a standard statistical speech segmentation task with one addition: word-onsets occurred in a small window around the onset of visual objects. Under these conditions, adults succeeded at both segmenting the speech stream, and mapping the words onto their correct referents (Cunillera et al., 2010a,b; Thiessen, 2010). However, in identical experiments, 8-month-olds failed to acquire either regularity (Thiessen, 2010). Further, when the task is made slightly more difficult – presenting multiple objects at once (as in Yu and Smith, 2007) – adults fail to learn word-object mappings from continuous speech (Frank et al., 2007). Thus, while parallel statistical learning might provide a significant advantage, it could be outside the processing limits of human learners (cf. Fiser and Aslin, 2002, for an example of parallel learning in a purely visual task). However, these demands on cognitive processing could be alleviated in another way: human learners could be scaffolded by other properties of natural language (Vygotsky, 1978; Mintz, 2003). The studies in this paper provide evidence for just such a solution in the context of parallel speech segmentation and word learning.

In typical statistical learning experiments, regularities in the input are constructed in such a way as to isolate the problem of interest. For instance, in statistical speech segmentation tasks, each word typically occurs with equal frequency and is equally likely to follow each other word (e.g., Saffran et al., 1996; Graf Estes et al., 2007). In statistical word learning tasks, each word and object typically occur with equal frequency, and each incorrect mapping has equal statistical support (e.g., Yu and Smith, 2007; Smith and Yu, 2008; Vouloumanos and Werker, 2009). But this structure differs in a number of ways from the structure of natural language input, and these difference are likely to matter (Kurumada et al., 2011; Vogt, 2012). For instance, referential utterances in child-directed speech often come from a small set of stereotyped naming frames, e.g., “*look at the dog*” (Cameron-Faulkner et al., 2003). Children are remarkably sensitive to this structure: 18-month-old infants orient faster to the referent of a label embedded in such statistically frequent naming frames than they do to a label uttered in isolation (Fernald and Hurtado, 2006). Do these frequent frames help learners segment a stream of sounds into *and* to map these words onto referents?

We pursued this question in two steps. First, we sought to determine the statistical structure of the frames that characterize naming events to young children. To this end, we analyzed data from a corpus of child-directed speech recorded during naturalistic free-play interactions to discover the shared structure of common naming frames. Subsequently, we constructed an artificial language in which the strings were naming events that maintained the main regularities found in the natural speech corpus. We then embedded these naming events in a word-object mapping task in which each trial contained multiple naming events and multiple visual referents. Thus, to learn the language, participants would have to segment labels from continuous speech *and* map them to their statistically consistent referents. We then parametrically manipulated the artificial language to determine if and how the regularities in natural naming frames facilitate simultaneous speech segmentation and word learning. Our findings illustrate the importance of understanding the statistical properties of natural language contexts for drawing conclusions about statistical learning.

## Results

### Corpus analysis

To capture regularities in naming frame structure, we analyzed transcripts of child-directed speech from naturalistic free-play interactions between 17 parent-child dyads (Yu et al., 2008; Yu and Smith, 2012). This corpus contained 3165 parental speech utterances, 1624 of which contained the label of one of the toys in the room. Of these utterances, 672 (∼20%) were single-word utterances consisting of only the toy’s label. Because the Experiments investigate the role of naming frames in parallel speech segmentation and word learning, these utterances were excluded from further analysis, but we return to them in the Discussion. The remaining 952 events were analyzed for consistent naming frame structure.

As shown in Table 1, 21 different naming frames cover more than 50% of all naming events. Together, these frames contain only 20 unique words and conform to two general regularities. First, in these frequent frames, the toy’s label always occurs in the final position (see also Aslin et al., 1996). Second, only a small set of words – mostly articles – precede a toy’s label (see also Shafer et al., 1998). Both regularities are also common in the remaining naming events, appearing in 50 and 63%, respectively. Because both final position (Endress et al., 2005) and onset cues (Bortfeld et al., 2005; Mersad and Nazzi, 2012) have previously been found to facilitate statistical sequence learning, each regularity could potentially scaffold statistical learners, buttressing them against the combinatorial explosion of parallel speech segmentation and word learning. Further, evidence from other studies suggests that redundant cues help children learn language (e.g., Gogate et al., 2000; Frank et al., 2009). Consequently the combination of both position and onset cues could play an additive role in speech segmentation and word learning.

**Table 1:** **The 21 most frequent naming frames**.

Phrase	Pct. of corpus
The OBJ	6.30
That is a OBJ	4.73
And the OBJ	4.31
A OBJ	4.10
It is a OBJ	3.78
This is a OBJ	3.57
And a OBJ	3.26
Can you say OBJ	2.94
Here is the OBJ	2.63
And OBJ	2.42
Where is the OBJ	1.89
That is the OBJ	1.79
Look at the OBJ	1.79
I have the OBJ	1.47
You want the OBJ	1.16
Color is the OBJ	1.16
Is that the OBJ	1.16
there is the OBJ	1.05
You put the OBJ	1.05
To put the OBJ	0.95
One is the OBJ	0.95
Total	52.42%

*Two regularities are apparent in the most frequent naming frames. First, Object Labels occur reliably in final frame position. Second, labels are reliably preceded by a small set of onset cues (a, the, and, say)*.

### Experiments

**Figure 1:**
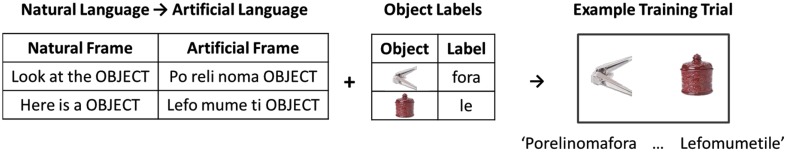
**An example training trial from the *Full* language condition**. Trials were constructed by encoding naming event patterns from the child-directed speech corpus into the artificial language.

To study joint speech segmentation and word-object mapping, we exposed adult participants to a series of individually ambiguous training trials based on the cross-situational learning paradigm (Yu and Smith, 2007). On each trial, adults saw two objects and heard two phrases of continuous speech from an artificial language. In order to learn word-object mappings, they had to determine which phrase referred to which object, where the word boundaries were, and finally which words were Object Labels and which word were Frame Words. Crucially, the naming frames extracted from the natural child-directed speech corpus were encoded into the artificial language presented to participants (Figure 1).

Participants were assigned randomly to one of four language conditions. In the *Full* language condition, participants heard artificial language phrases containing both regularities found in natural naming frames. In the *Onset Only* language condition, Object Labels appeared in the middle of phrases instead of at the end, but they were always preceded by one of a small set of onset cue words. In the *Position Only* language condition Object Labels always appeared in utterance-final position, but were not preceded by a small set of onset cue words. Finally, in the *Control* language condition, neither regularity from the natural naming frames was provided. After training, participants were tested for their knowledge of both the words of the language (speech segmentation), and the word-object mappings. Additional details can be found in the section “Materials and Methods” below.

#### Speech segmentation

**Figure 2:**
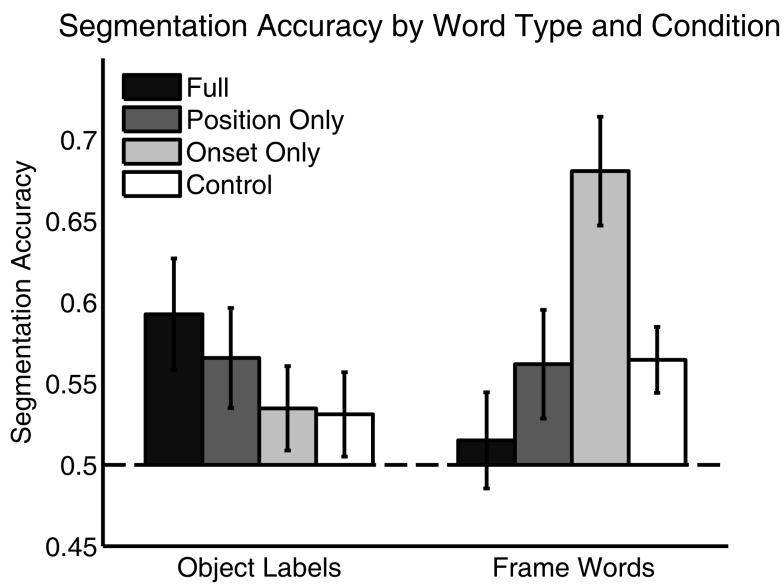
**Segmentation accuracy in each condition for both Object Labels and Frame words**. Learners successfully segmented Object Labels in the *Full* and *Position Only* language conditions, and segmented Frame Words in the *Onset Only* language condition. Error Bars indicate ±1 SE.

On each segmentation test, participants were asked to indicate which of two sequences was more likely to be a word of the language. Figure 2 shows how participants’ segmentation of both Object Labels and Frame Words varied across language conditions. Overall, participants successfully segmented Object Labels only in the *Full* and *Position Only* language conditions. They segmented Frame Words successfully in the *Onset Only* language condition, and to a lesser extent in the *Position Only* and *Control* language conditions. Participants’ segmentation accuracies were averaged across all words and submitted to a mixed 4 (Language) × 2 (Word Type) ANOVA. This analysis showed no main effect of language [*F*(3,90) = 1.40, *p* = 0.25] nor word type [*F*(1,90) = 0.83, *p* = 0.37], but did show a significant interaction [*F*(3,90) = 5.39, *p* < 0.01]. All segmentation accuracy were submitted to the Shapiro–Wilk test of normality (Shapiro and Wilk, 1965). Since none were found to be non-normal (all *p*’s > 0.1), follow up analyses used *t*-tests. These follow up tests showed that Object Label segmentation was above chance in the *Full* [*M* = 0.59, *t*(23) = 2.69, *p* < 0.05] and *Position Only* language conditions [*M* = 0.57, *t*(21) = 2.13, *p* < 0.05], but not in the *Onset Only* [*M* = 0.53, *t*(23) = 1.34, *p* = 0.19] or *Control* language conditions [*M* = 0.54, *t*(23) = 1.26, *p* = 0.22]. Frame-word segmentation was above chance in the *Onset Only* language condition [*M* = 0.68, *t*(23) = 5.39, *p* < 0.001], trended toward significance in the *Position Only* and *Control* language conditions [*M_PositionOnly_* = 0.56, *t*(21) = 1.86, *p* = 0.08; *M_Control_* = 0.55, *t*(21) = 1.93, *p* = 0.06] and was indistinguishable from chance in the *Full* language condition [*M* = 0.52, *t*(23) = 0.51, *p* = 0.62]. Segmentation of Object Labels and Frame Words was correlated in *Position Only* language condition (*r* = 0.48, *p* < 0.05), but not in any of the other language conditions (*r_Full_* = −0.22, *p* = 0.29; *r_OnsetOnly_* = 0.19, *p* = 0.39; *r_Control_* = 0.23, *p* = 0.29). Segmentation focus – and accuracy – thus varied across the conditions.

In the *Full* language condition, participants focused on and segmented only the Object Labels, learning little about the Frame Words. In the *Onset Only* language condition, participants segmented Frame Words very successfully, but failed to successfully segment the Object Labels. In the *Position Only* language condition, participants segmented Object Labels successfully and segmented Frame Words at near-significant levels. Further, segmentation accuracy for the two word types was correlated in this condition, suggesting that they supported each other. In the *Control* language condition, segmentation trended toward accuracy for the Frame Words and was at chance levels for Object Labels. Further, segmentation of the word types was uncorrelated, suggesting a less integrated segmentation strategy.

#### Word-object mapping

**Figure 3:**
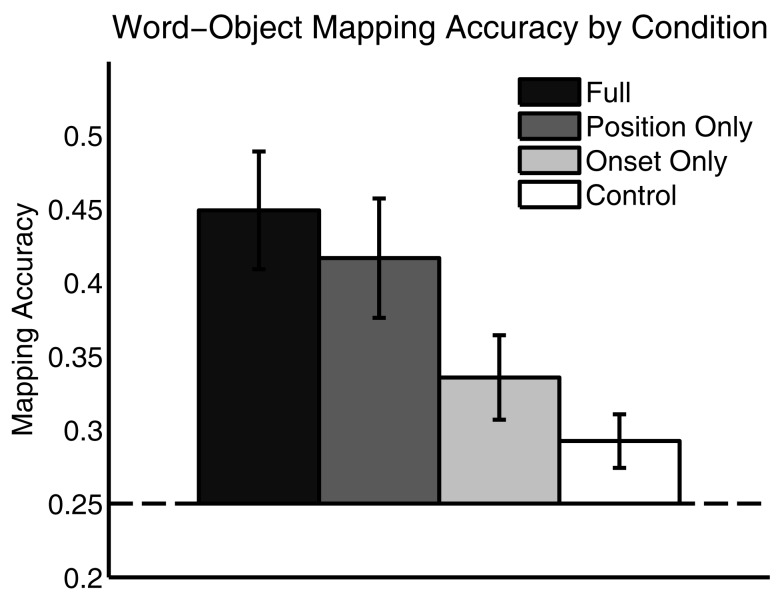
**Word-object mapping accuracy by condition**. Participants mapped words onto object successfully in all but the *Control* language condition. Error Bars indicate ±1 SE.

Participants were subsequently tested on their word-object mapping accuracy. On each test trial, they heard one word from training and were asked to select the most likely referent object from a set of four alternatives. As shown in Figure 3, participants learned a significant proportion of word-object mappings in all but the *Control* language condition, but were most successful in the *Full* and *Position Only* language conditions – the same languages in which they were most successful at Object Label segmentation. An ANOVA showed significant differences in mapping accuracy across conditions [*F*(3,90) = 5.03, *p* < 0.01]. Additional tests showed that accuracy was significantly above chance in all but the *Control* language condition [*M_Full_* = 0.45, *t*(23) = 4.98, *p* < 0.001; *M_PositionOnly_* = 0.42, *t*(21) = 4.12, *p* < 0.001; *M_OnsetOnly_* = 0.34, *t*(23) = 2.99, *p* < 0.01; *M_Control_* = 0.29, *t*(23) = 1.78, *p* = 0.09]. Further, accuracy was similar in the Full and *Position Only* language conditions [*t*(44) = 0.57, *p* = 0.57], and accuracy in both was significantly greater than in the *Control* language condition [*t_Full_*(46) = 3.69, *p* < 0.001; *t_PositionOnly_*(44) = 2.92, *p* < 0.01].Accuracy was significantly greater in the *Full* language condition than in the *Onset Only* language condition [*t*(46) = 2.31, *p* < 0.05], but accuracy did not differ between the *Position Only* and the *Onset Only* language conditions [*t*(44) = 1.65, *p* = 0.11]. Thus, participants were able to learn word-object mappings from continuous speech as long as either regularity from natural naming frames was present. However, the position regularity facilitated learning more than the onset cue regularity.

#### Correlations between speech segmentation and word-object mapping

**Figure 4:**
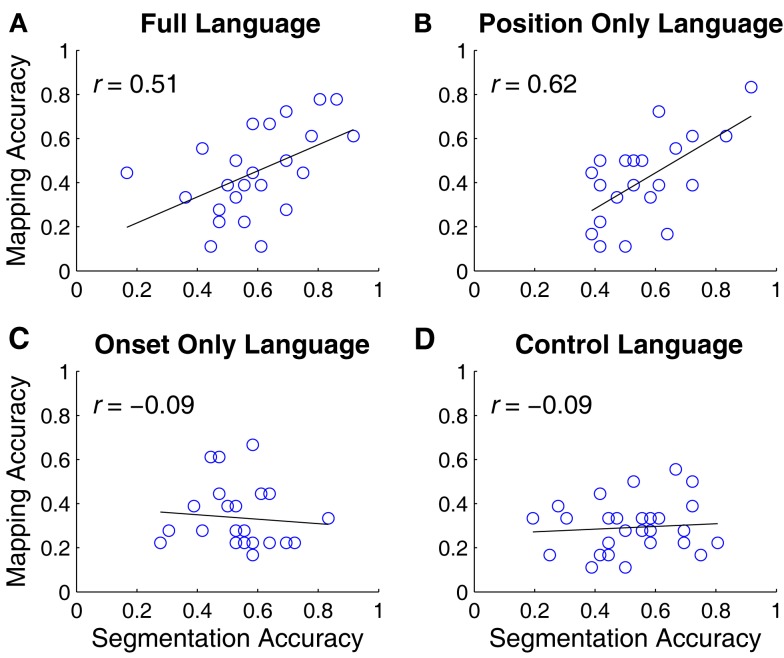
**Correlations between segmentation accuracy and word-mapping accuracy in each language condition**. Learning the two regularities was positively correlated in the *Full* **(A)** and *Position Only* **(B)** language conditions, and uncorrelated in the *Onset Only* **(C)** and *Control* **(D)** language conditions.

Did segmentation and word-object mapping interact, bootstrapping each other? Figure 4 shows correlations between each participant’s average Object Label segmentation and average word-object mapping in each language condition. The two were positively correlated in the *Full* (*r* = 0.51; *p* < 0.05) and the *Position Only* language conditions (*r* = 0.62, *p* < 0.01), but were uncorrelated in the *Onset Only* (*r* = −0.09, *p* = 0.67) and *Control* language conditions (*r* = −0.10, *p* = 0.56). Thus, participants in the *Onset Only* language condition showed evidence of learning word-object mappings without fully segmenting the labels from the utterances.

## Discussion

Natural languages are richly structured, containing regularities at multiple hierarchal levels. Statistical learning approaches to language acquisition typically focus on one level at a time, showing how the primitives from the level below can be used to construct the primitives for the level above. Alternatively, statistical language learning at every level could proceed in parallel, exploiting statistical redundancies across levels (Feldman et al., 2009; Johnson et al., 2010). On this account, a child learning a word-referent mapping may not need to wait until she has fully learned the word. But uncertainty at multiple levels imposes significant attention and memory demands on learners, demands that may prevent learning altogether (Frank et al., 2007; Thiessen, 2010). In this paper, we suggest that these demands may be alleviated by other regularities in natural language input, for instance, frequent naming frames (Mintz, 2003).

### Corpus analysis

Analyzing the structure of natural naming events is an important step toward modeling children’s word learning. Because consistency in naming event structure constrains the space of potential solutions, the same mechanism that fails in an unstructured environment may successfully extract words from fluent speech and map them to their referent objects when additional regularities are present. Our analysis showed, first, that a large proportion of naming events in naturalistic free-play are single-word utterances (see also Fernald and Morikawa, 1993; Brent and Siskind, 2001). These utterances could simplify later speech segmentation and give infants a leg up in later word learning (Brent and Siskind, 2001; Lew-Williams et al., 2011).

Second, our analysis revealed two regularities common to over 50% of naming events: labels occur in final phrasal position, and are preceded by an onset cue. We hypothesize that these regularities, like single-word utterances, could also scaffold statistical learning. Specifically, the information encoded in frequent naming frames may allow learners to identify the utterances most likely to be naming events and to spot the label within each frame, potentially without fully segmenting the other words. That is, word-referent mapping may begin before children know exact word boundaries (Yu et al., 2005).

### Experiments

Encoding these regularities into an artificial language, we tested this idea empirically. Exposing adult participants to artificial languages constructed from a corpus of child-directed speech, we were able to determine the independent and joint contributions of the two regularities apparent in the corpus. Keeping constant the words that make up naming phrases, we altered only their order across conditions. If parallel speech segmentation and word-object mapping rely on environmental cues to reduce cognitive load, this should be reflected in the learning rates across our four conditions.

In the *Full* language condition, which gave strong cues to the frame position of Object Labels as well as to their onset, participants successfully segmented labels from continuous speech and mapped them onto their referent objects. This success came in spite, or perhaps because, of chance-level performance on Frame Word segmentation. That is, participants were able to focus their attention on only the relevant portion of the speech steam (see also Cunillera et al., 2010a). These results, along with the strong correlation between word segmentation and word-object mapping, suggest that participants became attuned to the positional regularity and effectively ignored large portions of the speech input. This reduction in cognitive load may have supported learning.

The *Position Only* language condition, in contrast, removed the onset cue by moving words in the cue set to the beginning of each sentence. In this condition, participants also successfully segmented Object Labels from continuous speech, although at slightly a reduced level. In trade, they performed at a near-significant level on Frame Word segmentation. Also, unlike in the *Full* language condition, segmentation of Object Labels and Frame Words was highly correlated, suggesting an interaction between the processes. Nonetheless, despite these differences, participants in the *Position Only* language condition performed well on the test of word-object mapping. Thus, removing the onset cue forced participants to actively process more of the speech stream, but the presence of the position cue kept cognitive load low enough to enable learning. These results are consistent with previous work showing that utterance-final position facilitates language learning (Echols and Newport, 1993; Goodsitt et al., 1993; Endress et al., 2005; Frank et al., 2007).

Removing the position regularity from the *Full* language yielded the *Onset Only* language condition. In this condition, Object Labels were preceded by a small set of onset cues, but occurred always in medial phrasal position. Without labels in final position, participants performed at chance on tests of Object Label segmentation. However performance on Frame Word segmentation reached levels unseen in the other conditions. Surprisingly, although participants did not show knowledge of correct Object Label segmentation, they did succeed in mapping words to objects at above chance (albeit reduced) levels. Thus, an onset cue alone was sufficient to enable word learning. This is consonant with other work showing that familiar words can act as onset cues, giving infants a wedge into speech segmentation (Bortfeld et al., 2005; Mersad and Nazzi, 2012).

Finally, when naming phrases contained all of the same words but neither of the cues found in the child-directed speech corpus, participants showed poor learning of both kinds of statistics. Thus, in the *Control* language condition, participants were unable to cope with the cognitive load inherent in the simultaneous segmentation and word learning.

## Conclusion

We began by considering the relationship between statistical speech segmentation and statistical word learning. While previous work has demonstrated a serial link (e.g., Graf Estes et al., 2007; Mirman et al., 2008), in which word candidates generated via statistical segmentation are privileged in statistical word learning, a robust parallel demonstration has remained elusive (Frank et al., 2007; Thiessen, 2010). Perhaps the computational resources required by the tasks are simply too costly to allow their simultaneous resolution. We proposed that construction of previous artificial languages may have averaged out the very regularities that support a parallel solution in naturalistic environments. To borrow from J. J. Gibson, “it’s not [just] what is inside the head that is important, it’s what the head is inside of.”

Analysis of a corpus of child-directed speech from free-play found two potential sources of such scaffolding. First, Object Labels occurred consistently in the final position of naming phrases. Second, these labels were consistently preceded by one of a small set of onset cue words, predominantly articles. We constructed artificial languages following a 2 × 2 design to produce all possible presence/absence combinations of these regularities. Adult participants were exposed to an ambiguous word-object mapping task in the cross-situational word learning paradigm (Yu and Smith, 2007) in which labels were embedded within continuous speech phrases. These experiments allowed us to determine the independent and joint contributions of the two natural naming regularities. Although these studies use adult language learners as a proxy for child language learners (Gillette et al., 1999), future studies will need to ask this question more directly, using infant participants and measuring learning on-line over the course of training. This will allow finer-grained analysis of the relative time-course of acquisition of each regularity, making clearer whether learning is serial, parallel, or a mixture of both. Further, while the two major regularities found in the corpus have been observed in other corpora, further analyses will need to determine how naming frames change over development, and how these frames contribute to speech segmentation and word learning. Finally, it is important to know to what extent these kinds of frames characterize other languages. Although surely specific frames will differ from language to language, there are reasons to expect common regularities to generalize. For instance, Aslin et al. (1996) analyzed Turkish child-directed speech and found that mothers consistently placed target objects in final position even though this is ungrammatical.

These results highlight the importance of studying statistical language learning in the context of real language input. Although statistical learning is often studies under “unbiased” assumptions about input distributions (e.g., uniform word frequency), these assumptions can be a poor proxy for real-world input (e.g., Zipfian frequency). Sometimes, as in the *Full* language condition, natural input distributions facilitate statistical learning (see also, Johns and Jones, 2010; Kurumada et al., 2011). However, in other cases, natural input statistics make pure statistical learning difficult or impossible (e.g., Johnson and Tyler, 2010; Medina et al., 2011; Vogt, 2012). In such cases, we may be led to understand how other properties of the environment – or of children’s and adults’ perceptual systems – take up the slack. For instance, a number of previous studies highlight the importance of redundant information in language learning (e.g., Gogate et al., 2000, 2001; Frank et al., 2009; Goldstein et al., 2010; Grassmann and Tomasello, 2010; Smith et al., 2010; Riordan and Jones, 2011). In all of these cases, a difficult statistical language learning problem is made easier by the addition of redundant information, often information from a second sensory modality. For instance, the addition of a pointing (Grassmann and Tomasello, 2010) or synchronous motion (Gogate et al., 2000). This redundant information may make the regularity easier to notice. In other cases, this highlighting is accomplished with a single modality – e.g., presenting the label in a familiar voice (Bergelson and Swingley, 2012) or prosody (Thiessen et al., 2005; Shukla et al., 2011). Finally, in some cases this simplification may be accomplished by the child’s own perception/action system, which may act as a filter on the visual (Yurovsky et al., 2012; Yu and Smith, 2012).

Language learning is a process of navigating uncertainty, of leveraging partially learned regularities to learn other regularities (Gleitman, 1990; Smith, 2000). Consequently, there many many routes for breaking into language, and the route that learners adopt is likely to depend on the statistics in their input. For instance, in the *Full* language condition, participants learned word-object mappings by segmenting Object Labels but ignoring Frame Words. In contrast, participants in the *Position Only* language condition segmented both kinds of words, and participants in the *Onset Only* language condition learned word-object mappings but segmented only the Frame Words. In concert with previous research indicating that learners can ignore irrelevant statistical information (Cunillera et al., 2010a; Weiss et al., 2010), and focus on reliable statistical information (Smith, 2000; Colunga and Smith, 2005), these results present a picture of language acquisition as an adaptive process in which learners focus on and exploit the regularities most useful for the task at hand. Thus, the timing with which different regularities are acquired is likely to vary as a function of each learner’s input. There may thus be cases, as Peters (1977) suggested, in which children “learn the tune before the words.”

## Materials and Methods

All experiments reported in this paper were approved by the Human Subjects Office at the Indiana University Office of Research Administration. Informed consent was obtained from all participants prior to their participation in these experiments.

### Corpus analysis

#### Data

Transcripts of child-direct speech for naming frame analysis were drawn from free-play interactions between 17 mothers and their 17–19-month-old children. These dyads were seated across from each other and asked to play with three novel toys for 3 min at a time. They were given three such sets of toys, resulting in nine total minutes of interaction. Parents were taught labels for each of these toys (e.g., “*dax*,” “*toma*”) and asked to use these if they wished to refer to them by name. No other instructions were given.

Audio recordings of each parent’s speech were automatically partitioned into individual utterances using a threshold of 1 s of speech silence. This approach provides a consistent, objective cutoff and obviates the reliability issues involved in human coding. For the purpose of speech segmentation, the importance of utterance boundaries is that they provide salient stops that disambiguate word boundaries. Because previous research shows that pauses on the order of 100 ms (Ettlinger et al., 2011) and 400 ms (Finn and Hudson Kam, 2008) affect adult speech segmentation, and pauses on the order of 500 ms (Mattys et al., 1999) affect infant statistical speech segmentation, 1 s is a conservative estimate of the length of pauses that would provide disambiguating information to children.

These utterances were then transcribed by human coders into English. Naming frame regularities were extracted using a six-word window made up of three words on either side of a toy’s label. If fewer than three words preceded or followed a label in any given utterance, blanks were inserted to fill out the window (e.g., “_ _ the *toma* is blue _”). Next, individual toy labels were replaced with a common token (OBJ), and the frequency of each resulting multi-word frame was computed.

### Experiments

#### Participants

Ninety-two undergraduate students from Indiana University participated in exchange for course credit. All participants were self-reported native speakers of English. These participants were divided into four approximately equal groups, each exposed to one of the artificial languages.

#### Materials

Stimuli for the experiment consisted of 18 unique objects (from Yu and Smith, 2007), and 38 unique words. Eighteen of these words acted as labels for the novel objects, and the other 20 were mapped onto the words contained in the 21 most frequent frames found in the corpus analysis. Half of the words of each type were one syllable (CV) long, and the other half were two syllables (CVCV) long, necessitating the construction of 57 unique syllables. These syllables were created by sampling 57 of the 60 possible combinations of 12 constants and 5 vowels. Syllables were assigned to words randomly, so that nothing about a word’s phonetic properties could be used to distinguish Object Labels from Frame Words.

Words were then concatenated together without intervening pauses to create artificial language equivalents of each of the 21 frequent frames in the corpus. Participants were exposed to synthesized versions of these phrases constructed with MBROLA (Dutoit et al., 1996). This produced utterances in which no prosodic or phonetic properties could be used to determine word boundaries, forcing participants to rely on statistical information. Speech was synthesized using the *us*1 diphone database – an American female speaking voice. Each consonant was 94 ms long with a pitch point of 200 Hz at 10 ms. Each vowel was 292 ms long with a 221 Hz pitch point at 108 ms and a 200 Hz pitch point at 292 ms. Each syllable was separated from the next by a 1 ms pause and each utterance ended with a 20 ms pause. These values were chosen to produce speech with a natural sound and cadence.

#### Design and procedure

Participants were told that they would be exposed to scenes consisting of two novel objects, and a phrase referring to each of them. Each phrase would contain exactly one word labeling an on-screen object, along with several function words corresponding to the grammar of the artificial language. Participants had to determine which phrase referred to which object, how the phrases they heard should be segmented into words, and which of these words referred to which of the objects. Next, participants observed an example trial using English words and familiar objects to demonstrate the task. Importantly, the example contained both an object-final phrase (“observe the tractor”) and an object-medial phrase (“and the dog over there”) to prevent participants from expecting any particular positional regularity.

After the example, participants observed 108 training trials, each containing 2 objects and 2 spoken artificial language phrases (Figure 1). Trials began with 2 s of silence, each phrase was approximately 2 s in length, and 3 s of silence succeeded each phrase, resulting in trials approximately 12 s long. Each object appeared 12 times, and each naming frame occurred a number of times proportional to its appearance in the child-directed speech corpus. The entire training set ran just over 20 min.

After training, participants were tested first for speech segmentation and then word-object mapping. On each segmentation test trial, a participant heard 2 two-syllable words: a word from the experiment and a foil created by concatenating the first syllable of one word and the second syllable of another (following Fiser and Aslin, 2002). They were asked to indicate which of the words was more likely to be part of the artificial language (2AFC Test). Six correct Object Labels were tested against 6 Object foils, and 6 correct Frame Words were tested against 6 Frame foils, resulting in 72 total segmentation trials. Each possible word occurred an equal number of times in testing, preventing participants from using test frequency as a cue to correctness. Tests for Object Labels and Frame words were interspersed in a different random order for each participant.

Subsequently, participants were tested on their knowledge of word-object mappings. On each test trial, participants heard one of the Object Labels and were asked to select its correct referent from a set of four alternatives (4AFC Test). All of the labels were tested once in random order.

To assess the independent and joint contribution of both the final position and onset cue regularities, one group of participants was exposed to each of the four possible presence/absence combinations of these cues. Materials and procedure were identical for each of the groups except for the order of words within each artificial language naming phrase (Table 2). To quantify the in-principle difficulty of segmenting each language, we compute the binary entropy of the Frame Words in the positions preceding and following an Object Label in each language condition. Entropy (*H*) quantifies the variability of a distribution, integrating both the number of unique alternatives and the relative frequency of each alternative (Shannon, 1948). When there is no variability, e.g., when the only possibility is an utterance boundary, entropy is zero. As the number of alternatives increases and their frequencies become more uniform, entropy increases. Onset and Offset entropies for each language are also found in Table 2.

**Table 2:** **The 2 × 2 design of the artificial language experiment**.

	Final position	Middle position
Preceding cue	*Full Language* “Look at *the* OBJ” Onset *H*: 1.45, Offset *H*: 0	*Onset Only Language* “At *the* OBJ look” Onset *H*: 1.45, Offset *H*: 3.50
No cue	*Position Only Language* “*The* look at OBJ” Onset *H*: 2.71, Offset *H*: 0	*Control Language* “*the* look OBJ at” Onset *H*: 2.71, Offset *H*: 3.50

*Phrasal position of the Object Label varies along the rows; presence of the onset cue varies along the columns*.

## Conflict of Interest Statement

The authors declare that the research was conducted in the absence of any commercial or financial relationships that could be construed as a potential conflict of interest.
